# Anomalously high value of Coulomb pseudopotential for the H_5_S_2_ superconductor

**DOI:** 10.1038/s41598-018-30391-z

**Published:** 2018-08-10

**Authors:** Małgorzata Kostrzewa, Radosław Szczęśniak, Joanna K. Kalaga, Izabela A. Wrona

**Affiliations:** 1 Institute of Physics, Jan Długosz University in Częstochowa, Ave. Armii Krajowej 13/15, 42-200 Częstochowa, Poland; 2 Institute of Physics, Częstochowa University of Technology, Ave. Armii Krajowej 19, 42-200 Częstochowa, Poland; 3 Quantum Optics and Engineering Division, Faculty of Physics and Astronomy, University of Zielona Góra, Prof. Z. Szafrana 4a, 65-516 Zielona Góra, Poland

## Abstract

The H_5_S_2_ and H_2_S compounds are the two candidates for the low-temperature phase of compressed sulfur-hydrogen system. We have shown that the value of Coulomb pseudopotential (μ*) for H_5_S_2_ ([*T*_*C*_]_e*xp*_ = 36 K and *p* = 112 GPa) is anomalously high. The numerical results give the limitation from below to μ* that is equal to 0.402 (μ* = 0.589), if we consider the first order vertex corrections to the electron-phonon interaction). Presented data mean that the properties of superconducting phase in the H_5_S_2_ compound can be understood within the classical framework of Eliashberg formalism only at the phenomenological level (μ* is the parameter of matching the theory to the experimental data). On the other hand, in the case of H_2_S it is not necessary to take high value of Coulomb pseudopotential to reproduce the experimental critical temperature relatively well (μ* = 0.15). In our opinion, H_2_S is mainly responsible for the observed superconductivity state in the sulfur-hydrogen system at low temperature.

## Introduction

The Eliashberg formalism^1^ allows for the analysis of classical phonon-mediated superconducting state at the quantitative level. The input parameters to the Eliashberg equation are: the spectral function, otherwise called the Eliashberg function (*α*^2^*F*(*ω*)) that is modeling the electron-phonon interaction^2,3^, and the Coulomb pseudopotential (μ*), which is responsible for the depairing electron correlations^4,5^. Usually, the Eliashberg function is calculated using the DFT method (*e.g*. in the Quantum Espresso package^6,7^). The value of Coulomb pseudopotential is selected in such way that the critical temperature, determined in the framework of Eliashberg formalism, would correspond to *T*_*C*_ from the experiment. Sometimes μ* is tried to be calculated from the first principles, however, this is the very complex issue and it is rarely leading to correct results^8^.

It should be noted that the value of Coulomb pseudopotential should not exceed 0.2. For higher values, μ* cannot be associated only with the depairing electron correlations. In the case at hand, the quantity μ* should be treated only as the parameter of fitting the model to the experimental data, which means that the Eliashberg theory becomes practically the phenomenological approach.

The phonon-mediated superconducting state in the compressed H_2_S with high value of critical temperature was discovered by Drozdov, Eremets, and Troyan in 2014^9^ (see also the paper^10^). The experimental observation of superconductivity in the compressed dihydrogen sulfide was inspired by the theoretical prediction made by Li *et al*.^11^, which is based on the extensive structural study on the H_2_S compound at the pressure range of ~10–200 GPa. In the paper^11^ the following sequence of structural transitions was demonstrated: at the pressure of 8.7 GPa the *Pbcm* structure is transformed into the *P2/c* structure. The next transformation occurs for the pressure of 29 GPa (*P2/c* → *Pc*). For the pressure of 65 GPa the transformation of *Pc* structure into the *Pmc*2_1_ structure was observed. It is worth paying attention to the fact that the obtained theoretical results are consistent with the X-ray diffraction (XRD) experimental data^12,13^. The last two structural transitions for compressed H_2_S were observed for 80 GPa (*Pmc*2_1_ → *P-1*) and 160 GPa (*P-1* → *Cmca*). Interestingly, the results obtained in the paper^11^ are in the contradiction with the earlier theoretical predictions, suggesting that H_2_S dissociate into elemental sulfur and hydrogen under the high pressure^14^. However, it should be noted, that the partial decomposition of H_2_S was observed in Raman^15^ and XRD studies^16^ at the room temperature above 27 GPa. The calculations of the electronic structure made in^11^ suggest that the H_2_S compound is the insulator up to the pressure of 130 GPa. This result correlates well with the value of the metallization pressure of about 96 GPa, observed experimentally^17^ (see also related research^12,13,15,16,18–24^). It is worth noting that in the work^11^ for the structure of *Pbcm* (*p* = 0.3 GPa) the large indirect band gap of ~5.5 eV was determined, which is relatively good comparing with the experimental results (4.8 eV)^25^. With the pressure increase, the band gap decreases (3.75 eV for 15 GPa, 1.6 eV for 40 GPa, and 0.27 eV for 120 GPa). For the metallization pressure (130 GPa), the value of the electron density of states (*ρ*(*ε*_*F*_)) is 0.33 eV^−1^ per f.u. The sudden increase in *ρ*(*ε*_*F*_) to the value of 0.51 eV^−1^ per f.u. is observed at the structural transition *P-1* → *Cmca*^11^.

In experiments described in the papers^9^ and^10^ are observed two different superconducting states. In particular, superconductivity measured in the low-temperature range (l-T, sample prepared at *T* < 100 K), possibly relates to the H_2_S compound, as it is generally consistent with calculations presented in^11^ for solid H_2_S: both the value of *T*_*C*_ < 82 K and its pressure behavior. In addition, it was demonstrated theoretically^26^ that the experimental results could be reproduced accurately in the framework of classical Eliashberg equations, whereas the value of Coulomb pseudopotential is low (μ* ~ 0.15).

On the other hand, the result obtained by Ishikawa *et al*.^27^ suggest that in the narrow pressure range from 110 GPa to 123 GPa, the H_5_S_2_ compound, in which asymmetric hydrogen bonds are formed between H_2_S and H_3_S molecules, is thermodynamically stable and its critical temperature correlates well with experimental results^9,10^. However, it should be assumed that the anharmonic effects lower the theoretically determined value of the critical temperature by the minimum of about 20%. This assumption has some theoretical justification^28,29^, nevertheless in the paper^27^, it is not supported by the first-principles calculations.

It is also worth paying attention to the results contained in^30^, in which it is envisaged that the H_4_S_3_ is stable within the pressure range of 25–113 GPa. What is important, H_4_S_3_ coexists with fraction of H_3_S and H_2_S, at least up to the pressure of 140 GPa. The theoretical results correlate with XRD data, which confirm that above 27 GPa, the dihydrogen sulfide partially decomposes into S + H_3_S + H_4_S_3_. Nevertheless, H_4_S_3_ is characterized by the very low critical temperature (*T*_*C*_ = 2.2 K for μ* = 0.13), which suggests that kinetically protected H_2_S in samples prepared at low temperature is responsible for the observed superconductivity below 160 GPa^30^.

The superconductivity with the record high value of critical temperature (*T*_*C*_ = 203 K and *p* = 155 GPa), obtained for the sample prepared at high temperatures (h-T), relates to the decomposition of starting material above 43 GPa^31^ (see also^28,30,32,33^): 3H_2_S → 2H_3_S + S. We notice that the experimental values of critical temperature and its pressure dependencies are close to the values of *T*_*C*_ predicted theoretically by Duan *et al*.^31,34^ (*T*_*C*_ ∈ 〈191–204〉 K at 200 GPa), or later by Errea *et al*.^29^ for the cubic *Im-3m* structure with H_3_S stoichiometry. The physical mechanism underlying the superconductivity of H_3_S is similar to that in the MgB_2_ compound: metallization of covalent bonds. The main difference from the magnesium dibore is that the hydrogen mass is 11 times smaller than the mass of boron^32^. Considering the electron-phonon interaction, it was noted that in the case of H_3_S, the vertex corrections in the local approximation have the least meaning^35^. However, in the static limit with finite **q**, the vertex corrections change the critical temperature by −34 K^36^. The result above correlates well with the data for SiH_4_^37^. For the H_3_S compound, the thermodynamic parameters are also affected by the anharmonic effects. It was shown that for 200 GPa and 250 GPa, the anharmonic effects lower substantially the value of electron-phonon coupling constant^28,36^. It is worth noting that the pressure which increases above 250 GPa does not increase the critical temperature value in H_3_S^38^. However, we have recently shown that the value of *T*_*C*_ increases (242 K), if we use the sulfur isotope ^36^S^39^. Therefore, it can be reasonably supposed that it is possible to obtain the superconducting condensate close to the room temperature (see also related research^40^).

In the presented paper, we analyze precisely the thermodynamic properties of superconducting state in the H_5_S_2_ system^27^. According to what we have mentioned before this compound is thermodynamically stable in the narrow pressure range from 110 GPa to 123 GPa. As the part of the analysis, we will prove that for the pressure at 112 GPa, the superconducting state is characterized by the anomalously high value of μ* (also after taking into account the vertex corrections to the electron-phonon interaction). The above result is not consistent with the result obtained by Ishikawa *et al*.^27^, where ${\mu }^{\ast }\in \langle 0.13,0.17\rangle$. The paper contains also the parameter’s analysis of superconducting state, that is induced in the H_4_S_3_ compound^30^. The results obtained by us were compared with the results for the H_2_S compound, which the thermodynamic properties of superconducting state naturally explain the properties of low-temperature superconducting phase in the compressed hydrogen sulfide^11,26^.

## Formalism

Let us take into account the Eliashberg equations on the imaginary axis ($i=\sqrt{-1}$):

1 $$\begin{array}{ccc}{\phi }_{n} & = & \pi {k}_{B}T\sum _{m=-\,M}^{M}\,\frac{{\lambda }_{n,m}-{\mu }_{m}^{\ast }}{\sqrt{{\omega }_{m}^{2}{Z}_{m}^{2}+{\phi }_{m}^{2}}}{\phi }_{m}-A\frac{{\pi }^{3}{({k}_{B}T)}^{2}}{4{\varepsilon }_{F}}\\  &  & \times \sum _{m=-\,M}^{M}\sum _{m^{\prime} =-\,M}^{M}\frac{{\lambda }_{n,m}{\lambda }_{n,m^{\prime} }}{\sqrt{({\omega }_{m}^{2}{Z}_{m}^{2}+{\phi }_{m}^{2})({\omega }_{m^{\prime} }^{2}{Z}_{m^{\prime} }^{2}+{\phi }_{m^{\prime} }^{2})({\omega }_{-n+m+m^{\prime} }^{2}{Z}_{-n+m+m^{\prime} }^{2}+{\phi }_{-n+m+m^{\prime} }^{2})}}\\  &  & \times [{\phi }_{m}{\phi }_{m^{\prime} }{\phi }_{-n+m+m^{\prime} }+2{\phi }_{m}{\omega }_{m^{\prime} }{Z}_{m^{\prime} }{\omega }_{-n+m+m^{\prime} }{Z}_{-n+m+m^{\prime} }-{\omega }_{m}{Z}_{m}{\omega }_{m^{\prime} }{Z}_{m^{\prime} }{\phi }_{-n+m+m^{\prime} }],\end{array}$$

and

2 $$\begin{array}{ccc}{Z}_{n} & = & 1+\frac{\pi {k}_{B}T}{{\omega }_{n}}\sum _{m=-M}^{M}\frac{{\lambda }_{n,m}}{\sqrt{{\omega }_{m}^{2}{Z}_{m}^{2}+{\phi }_{m}^{2}}}{\omega }_{m}{Z}_{m}-A\frac{{\pi }^{3}{({k}_{B}T)}^{2}}{4{\varepsilon }_{F}{\omega }_{n}}\\  &  & \times \sum _{m=-M}^{M}\sum _{m^{\prime} =-M}^{M}\frac{{\lambda }_{n,m}{\lambda }_{n,m^{\prime} }}{\sqrt{({\omega }_{m}^{2}{Z}_{m}^{2}+{\phi }_{m}^{2})({\omega }_{m^{\prime} }^{2}{Z}_{m^{\prime} }^{2}+{\phi }_{m^{\prime} }^{2})({\omega }_{-n+m+m^{\prime} }^{2}{Z}_{-n+m+m^{\prime} }^{2}+{\phi }_{-n+m+m^{\prime} }^{2})}}\\  &  & \times [{\omega }_{m}{Z}_{m}{\omega }_{m^{\prime} }{Z}_{m^{\prime} }{\omega }_{-n+m+m^{\prime} }{Z}_{-n+m+m^{\prime} }+2{\omega }_{m}{Z}_{m}{\phi }_{m^{\prime} }{\phi }_{-n+m+m^{\prime} }-{\phi }_{m}{\phi }_{m^{\prime} }{\omega }_{-n+m+m^{\prime} }{Z}_{-n+m+m^{\prime} }]\mathrm{.}\end{array}$$

In the case when *A* = 1, the Eliashberg set was generalized to include the lowest-order vertex correction - scheme VCEE (**V**ertex **C**orrected **E**liashberg **E**quations)^41^. On the other hand (*A* = 0), we get the model without the vertex corrections: the so-called CEE scheme (**C**lassical **E**liashberg **E**quations)^1^. Note that in the considered equations, the momentum dependence of electron-phonon matrix elements has been neglected, which is equivalent to using the local approximation.

The individual symbols in equations (1 and 2) have the following meaning: *φ*_*n*_ = *φ*(*iω*_*n*_) is the order parameter function and *Z*_*n*_ = *Z*(*iω*_*n*_) represents the wave function renormalization factor. The quantity *Z*_*n*_ describes the renormalization of the thermodynamic parameters of superconducting state by the electron-phonon interaction^2^. This is the typical strong-coupling effect, because *Z*_*n*=1_ in the CEE scheme is expressed by the formula: ${Z}_{n=1}\sim 1+\lambda$, where *λ* denotes the electron-phonon coupling constant: $\lambda =2{\int }_{0}^{{\omega }_{D}}d\omega {\alpha }^{2}F(\omega )/\omega$, and *ω*_*D*_ is the Debye frequency. The order parameter is defined as the ratio: ${{\rm{\Delta }}}_{n}={\varphi }_{n}/{Z}_{n}$. Symbol *ω*_*n*_ is the Matsubara frequency: *ω*_*n*_ = *πk*_*B*_*T*(2*n* + 1), while $n$ is the integer. Let us emphasize that the dependence of order parameter on the Matsubara frequency means that the Eliashberg formalism explicitly takes into account the retarding nature of electron-phonon interaction. In the present paper, it was assumed *M* = 1100, which allowed to achieve convergent results in the range from *T*_0_ = 5 K to *T*_*C*_ (see also^42^).

The function ${\mu }_{n}^{\ast }$ modeling the depairing correlations has the following form: ${\mu }_{n}^{\ast }={\mu }^{\ast }\theta ({\omega }_{c}-|{\omega }_{n}|)$. The Heaviside function is given by *θ*(*x*) and *ω*_*c*_ represents the cut-off frequency. By default, it is assumed that ${w}_{c}\in \langle 3{w}_{D},10{w}_{D}\rangle .$

The electron-phonon pairing kernel is expressed by the formula:

3 $${\lambda }_{n,m}=2{\int }_{0}^{{\omega }_{D}}d\omega \frac{\omega }{{\omega }^{2}+4{\pi }^{2}{({k}_{B}T)}^{2}{(n-m)}^{2}}{\alpha }^{2}F(\omega ).$$

The higher order corrections are not included in equation (3). The full formalism demands taking into consideration the additional terms related to the phonon-phonon interactions and the non-linear coupling between the electrons and the phonons. This has been discussed by Kresin *et al*. in the paper^43^.

The Eliashberg spectral function is defined as:

4 $${\alpha }^{2}F(\omega )=\frac{1}{2\pi \rho ({\varepsilon }_{F})}\sum _{{\bf{q}}\nu }\,\delta (\omega -{\omega }_{{\bf{q}}\nu })\frac{{\gamma }_{{\bf{q}}\nu }}{{\omega }_{{\bf{q}}\nu }},$$

with:

5 $$\begin{array}{ccc}{\gamma }_{{\bf{q}}\nu } & = & 2\pi {\omega }_{{\bf{q}}\nu }\sum _{ij}\int \frac{{d}^{3}k}{{{\rm{\Omega }}}_{BZ}}|{g}_{{\bf{q}}\nu }({\bf{k}},i,j{)|}^{2}\\  &  & \times \delta ({\varepsilon }_{{\bf{q}},i}-{\varepsilon }_{F})\delta ({\varepsilon }_{{\bf{k}}+{\bf{q}},j}-{\varepsilon }_{F}),\end{array}$$

where *ω*_**q***ν*_ determines the values of phonon energies and *γ*_**q***ν*_ represents the phonon linewidth. The electron-phonon coefficients are given by *g*_**q***ν*_(**k**, *i*, *j*) and *ε*_**k**,*i*_ is the electron band energy (*ε*_*F*_ denotes the Fermi energy).

For the purpose of this paper, the spectral functions obtained by: Li *et al*.^11^ (H_2_S), Ishikawa *et al*.^27^ (H_5_S_2_), and Li *et al*.^30^ (H_4_S_3_), have been taken into account. The Eliashberg functions were calculated in the framework of Quantum Espresso code^6,7^.

## Results

### The pseudopotential parameter

**Figure 1:**
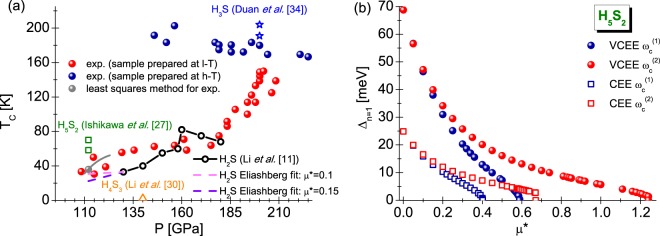
(**a**) The experimental values of critical temperature as a function of pressure for the selected hydrogen-containing compounds of sulfur. The following designation was concluded in the legend: exp. - experimental data taken from the paper^10^. The gray line represents the dependence of *T*_*C*_(*p*) in the pressure range from 110 GPa to 123 GPa, for which Ishikawa *et al*.^27^ predicted the stability of H_5_S_2_ compound. The black line with the circles indicates the theoretical results, obtained for H_2_S^11^. The violet and magenta dash lines are the predictions at the phenomenological level based on the classical theory of Eliashberg. In addition, we have included theoretical results for H_5_S_2_^27^ (green squares), H_4_S_3_^30^ (orange triangle) and H_3_S (blue stars)^34^. (**b**) The dependence of order parameter (Δ_*n*=1_) on the Coulomb pseudopotential for H_5_S_2_ - selected values of cut-off frequency (*T* = *T*_*C*_). The spheres represent the results obtained with the aid of Eliashberg equations with the vertex corrections (VCEE), the squares correspond to the results obtained in the framework of Migdal-Eliashberg formalism (CEE).

Figure 1(a) presents the experimental dependence of critical temperature on the pressure for the sulfur-hydrogen systems^10^ (see also^33^). Additionally, we included the theoretical results obtained for the H_5_S_2_^27^, H_2_S^11^, H_4_S_3_^30^, and H_3_S^34^.

It is worth to notice that H_5_S_2_ is thermodynamically stable in the fairly narrow pressure range (110–123 GPa). Especially at pressure 110 GPa there is the transformation: H_4_S_3_ + 7H_3_S → 5H_5_S_2_. On the other hand, breakup H_5_S_2_ for *p* = 123 GPa takes place according to the scheme: 3H_5_S_2_ → 5H_3_S + S.

The measuring points were used to determine the curve *T*_*C*_(*p*). We used the approximating function, matching the polynomial with the least squares method, respectively. On this basis, the estimated value of critical temperature for the pressure at 112 GPa is equal to 36 K. In the case of H_5_S_2_ compound with value of *T*_*C*_ = 36 K, we have calculated the pseudopotential parameter. To this end, we used the equation: ${[{{\rm{\Delta }}}_{n=1}({\mu }^{\ast })]}_{T={T}_{C}}=0$^44^. We obtained the very high value of μ* in both considered approaches: ${[{\mu }^{\ast }({\omega }_{c}^{(1)})]}_{VCEE}=0.589$ and ${[{\mu }^{\ast }({\omega }_{c}^{(1)})]}_{CEE}=0.402$, whereby we have chosen the following cut-off frequency: ${\omega }_{c}^{(1)}=3{\omega }_{D}$, where ${\omega }_{D}=237.2$ meV^27^. Additionally for H_5_S_2_, we have *ε*_*F*_ = 22.85 eV.

Note that the high value of μ* is relatively often observed in the case of high-pressure superconducting state. For example, for phosphorus: ${[{\mu }^{\ast }(5{\omega }_{D})]}_{CEE}^{p\mathrm{=20}{\rm{G}}Pa}=0.37$, where ${\omega }_{D}=59.4$ meV^45^. We encounter the similar situation for lithium: ${[{\mu }^{\ast }(3{\omega }_{D})]}_{CEE}^{p\mathrm{=29.7}{\rm{G}}Pa}=0.36$, while *ω*_*D*_ = 82.7 meV^46^. Interestingly, for H_3_S compound under the pressure at 150 GPa, the value of Coulomb pseudopotential is low: 0.123^47^. However, after increasing the pressure by 50 GPa, this value is clearly increasing: ${\mu }^{\ast }\sim 0.2$^35^. In the case of another compound (PH_3_), for which we also know the experimental critical temperature (*T*_*C*_ = 81 K), the much lower value of Coulomb pseudopotential was obtained: ${[{\mu }^{\ast }(10{\omega }_{D})]}_{CEE}^{p\mathrm{=200}{\rm{G}}Pa}=0.088$^35^. It is also worth mentioning the paper^44^, where the properties of superconducting state were studied in the SiH_4_ compound for ${\mu }^{\ast }\in \langle 0.1,0.3\rangle$. The result was the decreasing critical temperature range from 51.7 K to 20.6 K, what in relation to the results contained in the experimental work^48^ suggests ${\mu }^{\ast }\sim 0.3$. However, it should be remembered that the results presented in the publication^48^ are very much undermined^49^, while it is argued that the experimental data does not refer to SiH_4_ but to the PtH compound (the hydrogenated electrodes of measuring system).

Considering the facts presented above, it is easy to see that the H_5_S_2_ compound, even in the group of high-pressure superconductors, has the unusually high value of Coulomb pseudopotential. This is the feature of H_5_S_2_ system, because values of μ* cannot be reduced by selecting another acceptable cut-off frequency. On the contrary, increasing *ω*_*c*_ leads to the absurdly large increase in the value of Coulomb pseudopotential: ${[{\mu }^{\ast }({\omega }_{c}^{(2)})]}_{VCEE}=1.241$ and ${[{\mu }^{\ast }({\omega }_{c}^{(2)})]}_{CEE}=0.671$, where ${\omega }_{c}^{(2)}=10{\omega }_{D}$. The dependence courses of Δ_*n*=1_ on ${\mu }^{\ast }$, characterizing the situation discussed by us, are collected in Fig. 1(b).

It should be emphasized that value of μ* calculated by us for the CEE case (0.402) significantly exceeds the value of Coulomb pseudopotential estimated in the paper^27^, where ${\mu }^{\ast }\in \langle 0.13,0.17\rangle$. This result is related to the fact that in the publication^27^ the critical temperature was calculated using the simplified Allen-Dynes formula (*f*_1_ = *f*_2_ = 1)^50^, which significantly understates μ*, for values greater than 0.1^51^. This is due to the approximations used in the derivation of Allen-Dynes formula. Among other things, the analytical approach does not take into account the effects of retardation and the impact on the result of the cut-off frequency. Below is the explicit form of Allen-Dynes formula:

6 $${k}_{B}{T}_{C}={f}_{1}{f}_{2}\frac{{\omega }_{{\rm{l}}{\rm{n}}}}{1.2}\exp [\frac{-1.04(1+\lambda )}{\lambda -{\mu }^{\ast }(1+0.62\lambda )}],$$

where:

7 $${f}_{1}={[1+{(\frac{\lambda }{{{\rm{\Lambda }}}_{1}})}^{\frac{3}{2}}]}^{\frac{1}{3}},$$

8 $${{f}}_{2}=[1+\frac{(\frac{\sqrt{{{\omega }}_{2}}}{{{\omega }}_{\mathrm{ln}}}-1){{\lambda }}^{2}}{{{\lambda }}^{2}+{{\rm{\Lambda }}}_{2}^{2}}].$$

Parameters Λ_1_ and Λ_2_ were calculated using the formulas:

9 $${{\rm{\Lambda }}}_{1}=2.46(1+3.8{\mu }^{\ast }),$$

10 $${{\rm{\Lambda }}}_{2}=1.82(1+6.3{\mu }^{\ast })\frac{\sqrt{{\omega }_{2}}}{{\omega }_{{\rm{l}}{\rm{n}}}}\mathrm{.}$$

The second moment is given by the expression:

11 $${\omega }_{2}=\frac{2}{\lambda }{\int }_{0}^{{\omega }_{D}}d\omega {\alpha }^{2}F(\omega )\omega \mathrm{.}$$

The quantity *ω*_ln_ stands for the phonon logarithmic frequency:

12 $${\omega }_{\mathrm{ln}}=\exp [\frac{2}{\lambda }{\int }_{0}^{{\omega }_{D}}d\omega \frac{{\alpha }^{2}F(\omega )}{\omega }\,\mathrm{ln}(\omega )]\mathrm{.}$$

For the H_5_S_2_ compound, we have obtained: *ω*_2_ = 112.88 meV and *ω*_ln_ = 77.37 meV. Assuming *f*_1_ = *f*_2_ = 1, we reproduce the result contained in^27^:  ${[{{\rm{T}}}_{{\rm{C}}}]}^{{\mu }^{\ast }=0.17}=58.3$ K and ${[{{\rm{T}}}_{{\rm{C}}}]}^{{\mu }^{\ast }=0.13}=70.1$ K. However, the full Allen-Dynes formula gives: ${[{{\rm{T}}}_{{\rm{C}}}]}^{{\mu }^{\ast }=0.17}=62.5$ K and ${[{{\rm{T}}}_{{\rm{C}}}]}^{{\mu }^{\ast }=0.13}=76.1$ K.

From the physical point of view, the values of Coulomb pseudopotential calculated for H_5_S_2_, in the framework of full Eliashberg formalism, are so high that μ* cannot be associated only with the depairing Coulomb correlations. In principle, it should be treated only as the parameter to fit the model to the experimental data. Of course, there can be very much reasons that cause anomalously high value of μ*. The authors of paper^27^ pay special attention to the role of anharmonic effects. It should be emphasized that even if it were this way, the anharmonic nature of phonons should be taken into account not only in the Eliashberg function, but also in the structure of Eliashberg equations itself (this fact is usually omitted in the analysis scheme). In our opinion, the probable cause may also be non-inclusion in the Fröhlich Hamiltonian^52^ the non-linear terms of electron-phonon interaction^43^ or possibly anomalous electron-phonon interactions related to the dependence of full electron Hamiltonian parameters of system on the distance between the atoms of crystal lattice^53,54^.

However, in our opinion it is more likely that the experimental results obtained for the low temperature superconducting state of compressed sulfur-hydrogen system are largely related to the condensate being induced in the H_2_S compound (see black line in Fig. 1). In fact, the predicted thermodynamically stable pressure range of H_5_S_2_ is really narrow (110–123 GPa), and the inevitable kinetic barrier may prevent the decomposition of H_2_S into H_5_S_2_ in such narrow pressure range. It should also be mentioned that in the following two experiments^30^ and^33^, the XRD measurements on compressed H_2_S do not observe the formation of H_5_S_2_. On the other hand the XRD results in the paper^30^ indeed observed the residual H_2_S coexist with dissociation products H_3_S and H_4_S_3_ at least up to 140 GPa. It should also be emphasized that the anomalously high μ* obtained for H_5_S_2_ are extremely incompatible with the estimation of μ* value (0.1–0.13) contained in Ashcroft’s fundamental paper^55^ concerning the superconducting state in the hydrogen-rich compounds.

The value of Coulomb pseudopotential observed in H_5_S_2_ compound is so high that it is worth to confront it with other microscopic parameters obtained from *ab initio* calculations: (*ε*_*F*_ and *ω*_ln_)^27^. The most advanced formula on the Coulomb pseudopotential takes the following form^5^:

13 $${\mu }^{\ast }=\frac{\mu +a{\mu }^{2}}{1+\mu \,\mathrm{ln}\,(\frac{{\varepsilon }_{F}}{{\omega }_{\mathrm{ln}}})+a{\mu }^{2}\,\mathrm{ln}\,(\frac{\alpha {\varepsilon }_{F}}{{\omega }_{\mathrm{ln}}})},$$

where *a* = 1.38 and *α* ≃ 0.1. Symbol *μ* denotes product of the electron density of states at the Fermi level *ρ*(*ε*_*F*_) and the Coulomb potential *U* > 0. Note that the expression (13) is the non-trivial generalization of Morel-Anderson formula^4^:

14 $${\mu }^{\ast }=\frac{\mu }{1+\mu \,\mathrm{ln}(\frac{{\varepsilon }_{F}}{{\omega }_{\mathrm{ln}}})}\mathrm{.}$$

In particular, with the help of equation (13), it can be proven that the retardation effects associated with the electron-phonon interaction reduce the original value of *μ* to the value of μ*, but to the much lesser extent than expected by Morel and Anderson.

Using the formula (13), it is easy to show that the anomalously high value of μ* cannot result only from the strong electron depairing correlations. Namely in the limit *μ* → +∞, we obtain:  ${[{\mu }^{\ast }]}_{{\rm{\max }}}=\mathrm{1/}\mathrm{ln}(\alpha {\varepsilon }_{F}/{\omega }_{\mathrm{ln}})=0.295$. It is worth noting that the Morel-Anderson model is completely unsuitable for the analysis, because for μ* = 0.402 we get the negative (non-physical) value of *μ* = −0.48.

The value of Coulomb pseudopotential can also be tried to be estimated using the phenomenological Bennemann-Garland formula^56^:

15 $${\mu }_{{\rm{BG}}}^{\ast }\sim 0.26\rho ({\varepsilon }_{F})\mathrm{/[1}+\rho ({\varepsilon }_{F})\mathrm{].}$$

For the H_5_S_2_ compound subjected to the action of pressure at 112 GPa, we obtain ${\mu }_{{\rm{BG}}}^{\ast }=0.23$, which also proves the breakdown of the classical interpretation of μ*.

Taking into account all the facts given above, it is unlikely for the low-temperature phase of compressed sulfur-hydrogen to be tied with H_5_S_2_. The dependence of critical temperature on the pressure could be explained according to the H_2_S compound, whereas the low value of Coulomb pseudopotential is assumed (${\mu }^{\ast }=0.15$)^11,26^. In particular, let’s focus on the lowest pressure considered in^11^ (130 GPa). The calculations within the paper, are performed according to the CEE scheme and they give the critical temperature value of $\sim 30.6$ K^26^, which correlates well with the experimental value of *T*_*C*_ = 36 K for *p* = 112 GPa. It is worth noting that the electron-phonon interaction in H_2_S for the pressure of 130 GPa, is characterized by the following set of parameters: *λ* = 0.785, *ω*_ln_ = 81.921 meV, and $\sqrt{{\omega }_{2}}=112.507$ meV. Physically, this means the intermediate value of coupling between the electrons and phonons. H_5_S_2_ is the system characterized by the strong electron-phonon coupling (*λ* = 1.186). At the phenomenological level, one can even extend the *T*_*C*_(*p*) curve calculated in the paper^11^. For this purpose, with the help of the approximation function, we determined the relationship *λ*(*p*), which we used in the appropriately modified Eliashberg equations (the case of one-band equations presented in^57^). As the result for *p* = 112 GPa, we received ${T}_{C}\in \langle 30.7,22.3\rangle$ K assuming that ${\mu }^{\ast }\in \langle 0.1,0.15\rangle$. The full form of *T*_*C*_(*p*) curves, for ${\mu }^{\ast }=0.1$ and ${\mu }^{\ast }=0.15$ in the pressure range from 112 GPa to 130 GPa, is presented in Fig. 1(a).

It is also worth mentioning the superconducting state, which can potentially be induced in H_4_S_3_. The data obtained for the pressure of 140 GPa gives the following characteristics of the electron-phonon interaction^30^: *λ* = 0.42 and *ω*_ln_ = 71.869 meV (typical limit of weak coupling). Due to the low value of *λ*, the critical temperature can be calculated using the McMillan formula^58^ (*f*_1_ = *f*_2_ = 1). Assuming that ${\mu }^{\ast }=0.13$, we get *T*_*C*_ = 2.2 K. This result means that the superconducting state inducing in H_4_S_3_ cannot be equated with the low-temperature superconducting phase of compressed sulfur-hydrogen system. We probably have the analogous situation here as for the H_5_S_2_ compound, where kinetically protected H_2_S in samples prepared at low temperature is responsible for the observed superconductivity below 160 GPa^30^.

In the following chapters, for illustrative purposes, we calculated the thermodynamic parameters for the H_5_S_2_ compound. Note, that even for anomalously high value of μ*, the classical Eliashberg equations allow to set thermodynamic functions of superconducting state correctly, but at the phenomenological level^2,59^. The presented discussion is complemented by the results obtained in the CEE scheme for H_2_S (*p* = 130 GPa) and H_4_S_3_ (*p* = 140 GPa) compounds.

### The order parameter and the electron effective mass

**Figure 2:**
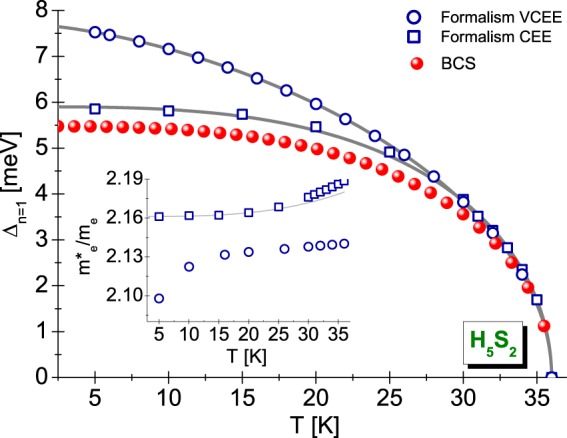
The influence of temperature on the maximum value of order parameter in the VCEE and CEE scheme for H_5_S_2_ (${\omega }_{c}={\omega }_{c}^{(1)}$). The insertion presents the ratio ${m}_{e}^{\ast }/{m}_{e}$ as a function of temperature. The open symbols represent the numerical results, the gray lines correspond to the results obtained with the help of analytical formulas ((16) or (17)). The red spheres were obtained in the BCS scheme assuming that: 2Δ_*n*=1_(0)/*k*_*B*_*T*_*C*_ = 3.53^62,63^.

Figure 2 presents for H_5_S_2_ the plot of the dependence of order parameter for the first Matsubara frequency on the temperature. It is clearly visible that, in the low temperature range, the values of order parameter calculated with regard to the vertex corrections are much higher than the values of Δ_*n*=1_ designated within the framework of classic Eliashberg scheme. This is the very interesting result, because the ratio *v*_*s*_ = *λω*_*D*_/*ε*_*F*_ for H_5_S_2_ compound is not high (*v*_*s*_ = 0.012)^27^. This result, with the cursory analysis, could suggest the slight influence of vertex corrections on the thermodynamic parameters. It is not, however, because it should be commemorated that *v*_*s*_ defines only the static criterion of the impact of vertex corrections. This means that the differences observed between the results obtained in the VCEE and CEE schemes are associated with dynamic effects, *i.e*. the explicit dependence of order parameter on the Matsubara frequency.

On the insertion in Fig. 2, we presented the influence of temperature on the value of the ratio of electron effective mass (${m}_{e}^{\ast }$) to the electron band mass (*m*_*e*_). In the Eliashberg formalism this ratio can be estimated using the following formula: ${m}_{e}^{\ast }/{m}_{e}={Z}_{n\mathrm{=1}}(T)$^2^. It can be seen that, in the entire temperature range analyzed by us, the effective mass of electron is high however slightly dependent on the temperature. In particular, in the VCEE scheme we get: ${[{m}_{e}^{\ast }]}_{{T}_{0}}=2.10{m}_{e}$ and ${[{m}_{e}^{\ast }]}_{{T}_{C}}=2.14{m}_{e}$. On the other hand, the classic Eliashberg approach gives: ${[{m}_{e}^{\ast }]}_{{T}_{0}}=2.16{m}_{e}$ and ${[{m}_{e}^{\ast }]}_{{T}_{C}}=2.19{m}_{e}$. When comparing the above results, we conclude that the vertex corrections have the very little effect on the value of the effective mass of electron, contrary to the situation with the order parameter.

The functions plotted in Fig. 2 can be characterized analytically by means of formulas:

16 $${{\rm{\Delta }}}_{n=1}(T)={{\rm{\Delta }}}_{n=1}(0)\sqrt{1-{(T/{T}_{C})}^{{\rm{\Gamma }}}}$$

and (only for the CEE scheme):

17 $${m}_{e}^{\ast }/{m}_{e}=[{Z}_{n=1}({T}_{C})-{Z}_{n=1}(0)]{(T/{T}_{C})}^{{\rm{\Gamma }}}+{Z}_{n=1}(0),$$

where the traditional markings were introduced: Δ_*n*=1_(0) = Δ_*n*=1_(*T*_0_) and *Z*_*n*=1_(0) = *Z*_*n*=1_(*T*_0_).

For the order parameter, we obtained the following estimation of temperature exponent: [Γ]_*VCEE*_ = 1.55 and [Γ]_*CEE*_ = 3.15. Physically, this result means that in the VCEE scheme the temperature dependence of order parameter differs very much from the course anticipated by the mean-field BCS theory, where Γ_BCS_ = 3^60^. On the other hand, not very large deviations from the predictions of BCS theory at the level of classical Eliashberg equations can be explained by referring to the impact of retardation and strong-coupling effects on the superconducting state. In the simplest way, they are characterized by the ratio *r* = *k*_*B*_*T*_*C*_/*ω*_ln_, which for the H_5_S_2_ compound equals 0.0401. On the other hand, in the BCS limit we obtain: *r* = 0. Of course, in the case of the VCEE scheme, the deviations from the BCS predictions should be interpreted as the cumulative effect of vertex corrections, strong-coupling, and retardation effects influencing the superconducting state.

**Figure 3:**
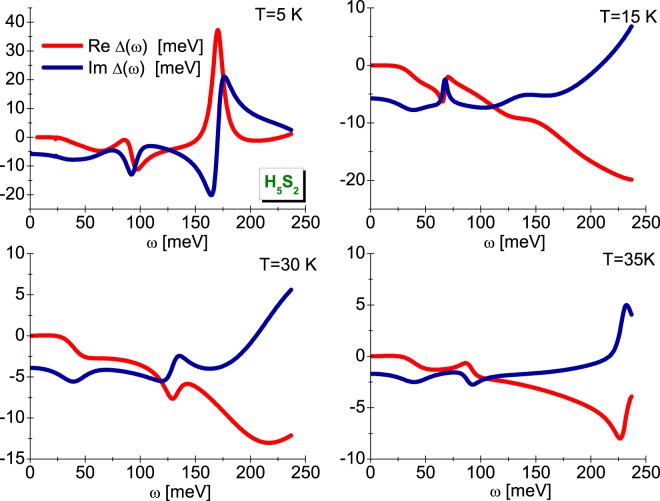
The real and imaginary part of H_5_S_2_ order parameter on the real axis for the selected values of temperature. The sample results obtained in the CEE scheme.

**Figure 4:**
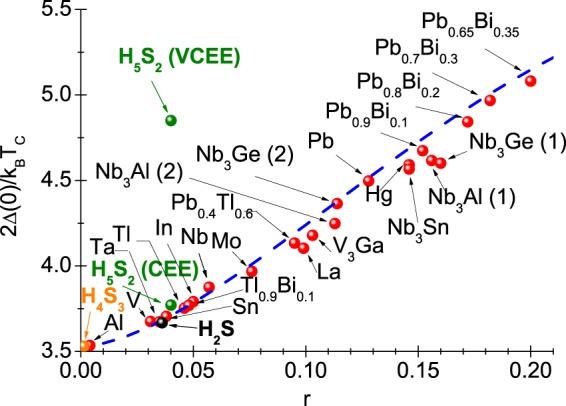
The value of ratio 2Δ(0)/*k*_*B*_*T*_*C*_ in the dependence on the parameter *r* = *k*_*B*_*T*_*C*_/*ω*_ln_. The blue line represents the general trend reproduced by the formula: 2Δ(0)/*k*_*B*_*T*_*C*_ = 3.53[1 + 12.5(*r*)^2^ln(1/2*r*)] (CEE scheme^2^).

The very accurate values of order parameter can be calculated by referring to the equation: Δ(*T*) = Re[Δ(*ω* = Δ(*T*))], where the symbol Δ(*ω*) denotes the order parameter determined on the real axis. The form of function Δ(*ω*) should be determined using the method of the analytical extension of imaginary axis solutions^61^. The sample results have been posted in Fig. 3, where it can be immediately noticed that the function of order parameter takes the complex values. However, for low values of frequency, only the real part of order parameter is non-zero. Physically, this means no damping effects, or equally, the endless life of Cooper pairs. Having the open form of order parameter on the real axis, we have calculated the value of ratio: *R*_Δ_ = 2Δ(0)/*k*_*B*_*T*_*C*_. The obtained result was compared with the data for other superconductors with the phonon pairing mechanism (see Fig. 4). It is easy to see that the result of CEE very well fits in the general trend anticipated by the classic Eliashberg formalism (*R*_Δ_ = 3.77). On the other hand, the impact of vertex corrections on the value of parameter *R*_Δ_ is strong (*R*_Δ_ = 4.85). Let us recall that the mean-field BCS theory predicts: *R*_Δ_ = 3.53^62,63^.

Particularly interesting is the comparison between the calculated values of *R*_Δ_ for H_5_S_2_ with results obtained for H_2_S and H_4_S_3_. In the first step, it is noticeable that the value of parameter *r* for H_2_S and H_4_S_3_ is 0.0362 and 0.0014, respectively. It is evident that ${[r]}_{{{\rm{H}}}_{2}{\rm{S}}}$ is close to the value of *r* obtained for H_5_S_2_, while in the case of H_4_S_3_ the retardation and strong-coupling effects do not play the major role. In the CEE scheme, the dimensionless ratio *R*_Δ_ for H_2_S and H_4_S_3_ assumes the values: ${[{R}_{{\rm{\Delta }}}]}_{{{\rm{H}}}_{2}{\rm{S}}}=3.67$ and ${[{R}_{{\rm{\Delta }}}]}_{{{\rm{H}}}_{4}{{\rm{S}}}_{3}}=3.53$. For the H_2_S and H_4_S_3_ compounds, the vertex corrections do not change *R*_Δ_. They do not influence the function Δ(*T*), which is why they are not taken into account in the further part of paper.

### The free energy, thermodynamic critical field, entropy, and the specific heat jump

The thermodynamics of superconducting state is fully determined by the dependence of order parameter on the temperature. Based on the results posted in Fig. 2, it can be seen that in the temperature area ${T}_{C}-T\ll {T}_{C}$, the values of Δ(*T*) obtained for H_5_S_2_ in the framework of VCEE and CEE schemes are physically indistinguishable. This result means that the thermodynamics of superconducting state near the critical temperature can be analyzed with the help of classical Eliashberg approach without vertex corrections.

The free energy difference between the superconducting and normal state has been calculated in agreement with the formula^64^:

18 $$\begin{array}{c}\frac{{\rm{\Delta }}F}{\rho \mathrm{(0)}}=-\,2\pi {k}_{B}T\sum _{m\mathrm{=1}}^{M}[\sqrt{{\omega }_{m}^{2}+{({{\rm{\Delta }}}_{m})}^{2}}-|{\omega }_{m}|]\\ \,\,\,\times \,[{Z}_{m}^{S}-{Z}_{m}^{N}\frac{|{\omega }_{m}|}{\sqrt{{\omega }_{m}^{2}+{({{\rm{\Delta }}}_{m})}^{2}}}],\end{array}$$

whereas ${Z}_{m}^{N}$ and ${Z}_{m}^{S}$ denote respectively the wave function renormalization factor for the normal state ($N$) and for the superconducting state (*S*).

**Figure 5:**
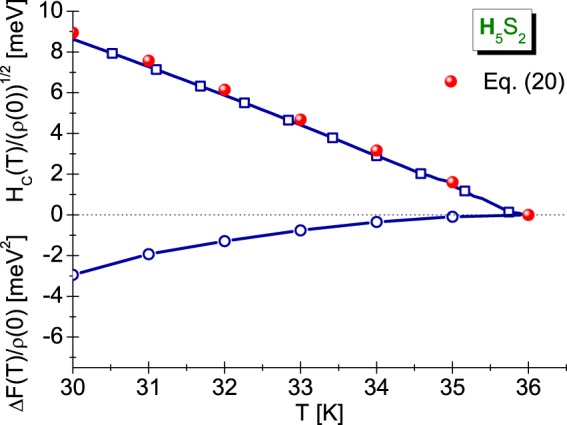
(lower panel) The free energy difference between the superconducting and normal state as a function of temperature, and (upper panel) the thermodynamic critical field. The charts were obtained as the part of classical Eliashberg formalism without the vertex corrections (for the temperature area in which the results obtained with the help of VECC and CEE models are identical (*T* > 30 K)).

In Fig. 5 (lower panel), we have plotted the form of function Δ*F*(*T*)/*ρ*(0) for H_5_S_2_. It can be seen that the free energy difference takes negative values in the whole temperature range up to T_C_. This demonstrates thermodynamic stability of superconducting phase in the compound under investigation. For the lowest temperature taken into account, it was obtained: [Δ*F*/*ρ*(0)]_*T*=30 K_ = −2.94 meV^2^.

On the basis of the temperature dependence of free energy difference, it is relatively easy to determine the values of thermodynamic critical field (*H*_*C*_), the difference in entropy (Δ*S*) between the superconducting state and the normal state, and the specific heat difference (Δ*C*).

The thermodynamic critical field has been calculated on the basis of formula:

19 $$\frac{{H}_{C}}{\sqrt{\rho (0)}}=\sqrt{-8\pi \frac{\Delta F}{\rho (0)}}\mathrm{.}$$

We presented the results obtained for the H_5_S_2_ compound in Fig. 5 (upper panel). We see that as the temperature rises, the critical field decreases so that in *T* = *T*_*C*_ its value equaled zero. Assuming for the H_5_S_2_ compound the following designation *H*(0) = *H*(*T* = 30 K) = 8.95 meV, it is easy to see that the critical field decreases in the proportion to the square of temperature, which is well illustrated by the parabola plotted on the basis of equation^65^:

20 $${H}_{C}(T)=H(0)[1-{(T/{T}_{C})}^{2}]\mathrm{.}$$

The dependence *H*_*C*_(*T*) determined using this formula is represented by the red spheres in Fig. 5.

The difference in the entropy between the superconducting and normal state has been estimated based on the expression:

21 $$\frac{{\rm{\Delta }}S}{{k}_{B}\rho (0)}=-\,\frac{d[{\rm{\Delta }}F/\rho (0)]}{d({k}_{B}T)}\mathrm{.}$$

**Figure 6:**
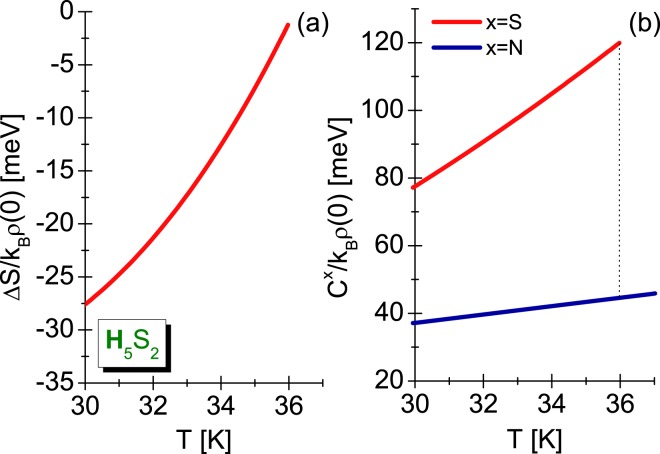
(**a**) The dependence of difference in the entropy on the temperature, and (**b**) the specific heat in the superconducting and normal state (the Eliashberg formalism without the vertex corrections).

We presented the obtained results in Fig. 6(a). Physically increasing are the value of entropy up to *T*_*C*_ proves the higher ordering of superconducting state in relation to the normal state.

The values of Δ*C* have been calculated using the formula:

22 $$\frac{{\rm{\Delta }}C}{{k}_{B}\rho (0)}=-\,\frac{1}{\beta }\frac{{d}^{2}[{\rm{\Delta }}F/\rho (0)]}{d{({k}_{B}T)}^{2}}\mathrm{.}$$

In addition, the specific heat of normal state is determined based on:

23 $$\frac{{C}^{N}}{{k}_{B}\rho \mathrm{(0)}}=\gamma {k}_{B}T,$$

where the Sommerfeld constant equals: $\gamma =\frac{2}{3}{\pi }^{2}\mathrm{(1}+\lambda )$. The influence of temperature on the specific heat in the superconducting state and the normal state has been presented in Fig. 6(b). The characteristic specific heat jump visible in the critical temperature is noteworthy. For the H_5_S_2_ compound its value equals 75.34 meV.

**Figure 7:**
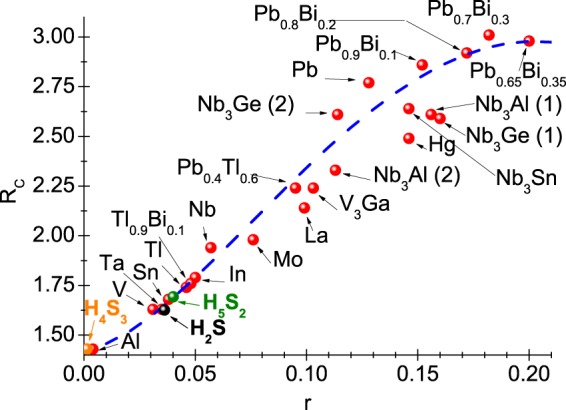
The value of ratio *R*_*C*_ in the dependence on the parameter *r*. The blue line represents the general trend obtained using the formula: *R*_*C*_ = 1.43[1 + 53(*r*)^2^ln(1/3*r*)] (CEE scheme^2^).

The thermodynamic parameters determined allow to calculate the dimensionless ratio: *R*_*C*_ = Δ*C*(*T*_*C*_)/*C*^*N*^(*T*_*C*_). As part of classic BCS theory, the quantity *R*_*C*_ takes the universal value equal to 1.43^62,63^. In the case of H_5_S_2_ compound, it was obtained *R*_*C*_ = 1.69. Hence, the value of *R*_*C*_ for H_5_S_2_ deviates from the predictions of BCS theory. Additionally, Fig. 7 presents the dependence of dimensionless parameter *R*_*C*_ on *r*. The chart shows that the value of *R*_*C*_ obtained for H_5_S_2_ perfectly fits the general trend anticipated by the classic Eliashberg formalism.

In the case of H_2_S compound the ratio *R*_*C*_ = 1.63 is close to ${[{R}_{C}]}_{{{\rm{H}}}_{{\rm{5}}}{{\rm{S}}}_{{\rm{2}}}}$. The parameter *R*_*C*_ for H_4_S_3_ is equal to the value predicted by the BCS theory.

## Summary

We have calculated the thermodynamic parameters of superconducting state for the H_5_S_2_ compound under the pressure at 112 GPa. We have solved the Eliashberg equations on the imaginary axis, both including the first-order vertex corrections to the electron-phonon interaction, as well as without vertex corrections. For both cases, we have obtained anomalously high values of Coulomb pseudopotential: ${[{\mu }^{\ast }({\omega }_{c}^{(1)})]}_{VCEE}=0.589$ and ${[{\mu }^{\ast }({\omega }_{c}^{(1)})]}_{CEE}=0.402$, while ${\omega }_{c}^{(1)}=3{\omega }_{D}$. Note that increase in the cut-off frequency only increases value of ${\mu }^{\ast }$ (*e.g*. for VCEE case ${\mu }^{\ast }=1.241$, where ${\omega }_{c}^{(2)}=10{\omega }_{D}$). The calculated values of ${\mu }^{\ast }$ proves that this parameter cannot be associated only with the depairing Coulomb correlations - in principle, it should be treated as the effective parameter of fitting the model to experimental data. The above results mean that it is very unlikely that the low-temperature superconducting state of compressed sulfur-hydrogen system is induced in the H_5_S_2_ compound. In our opinion, experimentally was observed the superconducting state in the H_2_S compound, which is kinetically protected in the samples prepared at the low temperature^11,30^. It should be emphasized that in the case of H_2_S reproducing the experimental dependence of critical temperature on the pressure does not require anomalously high value of Coulomb pseudopotential^26^.

In our paper, we also analyzed the thermodynamic properties of superconducting state in the H_4_S_3_ compound. It is characterized by the very low value of critical temperature (${[{T}_{C}]}_{{\rm{\max }}}\sim 2$ K)^30^, and it cannot be associated with the low temperature superconducting state of compressed dihydrogen sulfide. Probably, we have the analogous situation here like for the H_5_S_2_ compound, where kinetically protected H_2_S in the samples prepared at the low temperature is responsible for the observed superconductivity.

As part of the analysis, we calculated the thermodynamic parameters of superconducting state for the H_5_S_2_, H_2_S, and H_4_S_3_ compounds. In the case of H_5_S_2_, we have shown that the first order vertex corrections to the electron-phonon interaction play the important role, *i.e*. they significantly change the thermodynamics of superconducting state in the low temperature range, while nearby *T*_*C*_ they are practically irrelevant. For this reason, the dimensionless parameter *R*_Δ_ in the VCEE scheme is equal to 4.85, and in the CEE scheme it equals 3.77. On the other hand, for both cases it was obtained *R*_*C*_ = 1.693. The above values prove that the superconducting state in the H_5_S_2_ compound is not the state of BCS type. The superconducting state for H_2_S has the thermodynamic parameters with values are close to the ones determined for H_5_S_2_ in the CEE scheme. In particular, we got: *R*_Δ_ = 3.67 and *R*_*C*_ = 1.626. On the other hand, the superconducting state of H_4_S_3_ compound is the BCS type.

Regarding the results presented by Ishikawa *et al*.^27^, in respect to the Eliashberg function (harmonic limit), it should be assumed that these are the results proving the impossibility of correct description of the low-temperature superconducting phase of compressed sulfur-hydrogen system.
